# Integrated Assessment of Pb(II) and Cu(II) Metal Ion Phytotoxicity on *Medicago sativa* L., *Triticum aestivum* L., and *Zea mays* L. Plants: Insights into Germination Inhibition, Seedling Development, and Ecosystem Health

**DOI:** 10.3390/plants12213754

**Published:** 2023-11-02

**Authors:** Ionela-Catalina Vasilachi-Mitoseru, Vasile Stoleru, Maria Gavrilescu

**Affiliations:** 1 Department of Environmental Engineering and Management, “Cristofor Simionescu” Faculty of Chemical Engineering and Environmental Protection, “Gheorghe Asachi” Technical University of Iasi, 73 Prof. D. Mangeron Blvd., 700050 Iasi, Romania; ionela.vasilachi@yahoo.com; 2 Department of Horticultural Technologies, Faculty of Horticulture, “Ion Ionescu de la Brad” University of Life Sciences, 3 Mihail Sadoveanu Alley, 700490 Iasi, Romania; vstoleru@uaiasi.ro; 3 Academy of Romanian Scientists, 3 Ilfov Street, 050044 Bucharest, Romania

**Keywords:** copper, germination, lead, *Medicago sativa* L., phytoremediation, *Triticum aestivum* L., *Zea mays* L.

## Abstract

Environmental pollution with heavy metals has become a problem of major interest due to the harmful effects of metal ions that constantly evolve and generate serious threats to both the environment and human health through the food chain. Recognizing the imperative need for toxicological assessments, this study revolves around elucidating the effects of Pb(II) and Cu(II) ions on three plant species; namely, *Medicago sativa* L., *Triticum aestivum* L., and *Zea mays* L. These particular species were selected due to their suitability for controlled laboratory cultivation, their potential resistance to heavy metal exposure, and their potential contributions to phytoremediation strategies. The comprehensive phytotoxicity assessments conducted covered a spectrum of critical parameters, encompassing germination inhibition, seedling development, and broader considerations regarding ecosystem health. The key metrics under scrutiny included the germination rate, the relative growth of root and stem lengths, the growth inhibition index, and the tolerance index. These accurately designed experiments involved subjecting the seeds of these plants to an array of concentrations of PbCl_2_ and CuCl_2_ solutions, enabling an exhaustive evaluation of the phytotoxic potential of these metal ions and their intricate repercussions on these plant species. Overall, this study provides valuable insights into the diverse and dynamic responses of different plant species to Pb(II) and Cu(II) metal ions, shedding light on their adaptability and resilience in metal-contaminated environments. These findings have important implications for understanding plant–metal interactions and devising phytoremediation strategies in contaminated ecosystems.

## 1. Introduction

Heavy metals are metallic elements that have a relatively high density and are known for their toxic effects on living organisms when they accumulate in the environment. They are considered inorganic, persistent soil pollutants because they do not break down easily in the environment and can persist for extended periods. Instead, they tend to accumulate over time, potentially reaching levels that are harmful to ecosystems and human health [1,2].

Contamination of soil with heavy metals can lead to various adverse effects, including reduced plant growth and crop yields, contamination of food crops, and risks to wildlife and human populations. Understanding their impacts, implementing regulatory measures, and conducting ongoing research are essential steps in managing the risks associated with heavy metal contamination in soil [3,4,5].

### 1.1. Understanding Heavy Metals in the Environment: Justifying the Need for Investigating Their Impact

**Figure 1:**
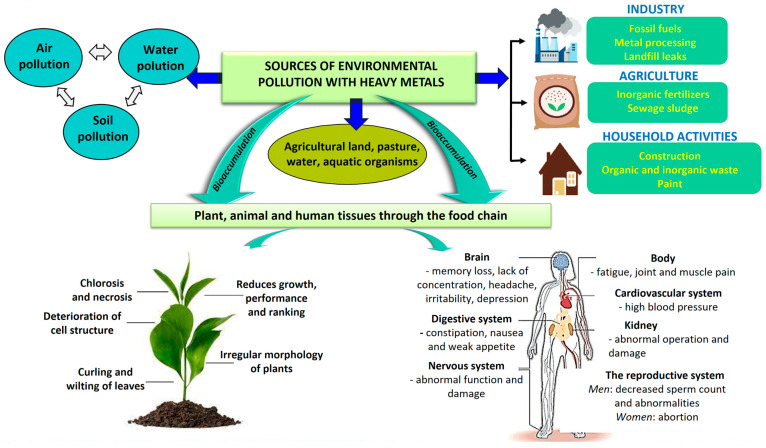
Sources and effects of heavy metals.

The sustainable development of human society is determined by the current and future management of its natural resources, with the aim to limit harm to plants. Metals such as mercury, cadmium, lead, and arsenic are not essential for plants and the environment in relation to objectives such as economic growth and ensuring the best possible quality of life and environment. Environmental pollution has become one of the most important and debated issues today. Along with industrialization and urbanization, the process of the degradation of environmental components has had an increasingly worrying evolution. Contamination of the environment with heavy metals is considered a major problem at the international level due to the harmful effects of these contaminants, leading to serious ecological and human problems through their accumulation in the food chain (Figure 1) [6,7,8].

Heavy metals such as cadmium (Cd), chromium (Cr), copper (Cu), mercury (Hg), lead (Pb), nickel (Ni), zinc (Zn), and arsenic (As) are included in the category of pollutants known as persistent inorganics (PIPs), which are used in agriculture and industry, and are therefore released into the environment. Consequently, at the European level, there are more than 10,000 sites contaminated with heavy metals [1,9].

Both natural and anthropogenic processes cause the occurrence of heavy metals in different components of the environment [10,11]. Some heavy metals are poisonous to plants, with negative effects even in low concentrations of metal ions, while other plants can accumulate heavy metals in their tissues to abnormal states without obvious side effects or yield reduction. Among the heavy metals, copper, boron, molybdenum, zinc, iron, and nickel are essential for plant growth, but also occur at concentrations above the permissible growth [4,12]. Anthropogenic activities—namely, industrial activities, mining, ore processing, and urbanization—have led to an increase in heavy metal concentrations in the environment. Metals such as Co, Cr, Cu, Ni, Cd, and Pb are considered an “environmental health hazard”; these pollutants [13,14,15] have been included in the priority list of dangerous substances, in the first ten positions, by the Agency for the Register of Toxic Substances and Medicines [16,17,18].

Among the most important characteristics of heavy metals are their toxicity, biodegradability, bioaccumulation, mobility, solubility, and oxidation number, which depend on the specific physico-chemical form in which the metal is found [19,20,21]. Many studies show that the free hydrate metal ion is the most toxic form, which is caused by its rapid absorption by the cells of organisms [22,23,24]. The toxicity of heavy metals in the soil depends on the concentrations detected (the maximum allowed limits differ from metal to metal), but also on the plant species. For example, there are metal-tolerant plants, called hyperaccumulating plants, with barrier mechanisms that can avoid or mitigate the harm caused by heavy metals [12]. Shoots and roots are physiologically, functionally, and spatially distinct organs. The root is the first organ that comes into contact with the metal-contaminated soil. This restricts the entry of heavy metals by undergoing changes such as lignification [25]. Also, heavy metals in excess have mutagenic or even carcinogenic properties, which cause irreversible processes in organisms (Figure 1) [26].

### 1.2. Impact of Heavy Metals on Cereal Crop Growth and Quality

The global cereal production was reported at about 3072 million metric tons in 2021. Wheat, rice, and maize (corn) are the three major cereal crops accounting for the majority of global cereal consumption. The surge in demand for cereals for both food and animal feed purposes is poised to be driven predominantly by Asian countries, marking a significant shift in global consumption patterns. Forecasts indicate a gradual uptick in global cereal utilization, projected to ascend from 2.8 billion metric tons in the baseline period (2022–2023) to approximately 3.1 billion metric tons by the year 2031 [27,28,29,30].

The escalating global appetite for cereals in animal feed applications is projected to be led by maize, forecasted to experience an annual growth rate of 1.3%, followed by wheat at 0.8%, and other coarse grains at 0.7% over the forthcoming decade. The demand for wheat for consumption is projected to witness a 20 million metric ton increase. This trend is driven by a growing preference for processed wheat products, including pastries and noodles. These processed goods necessitate higher quality, protein-rich wheat, a category predominantly supplied by countries such as the United States, Canada, Australia, and, to a lesser extent, the countries within the European Union [28,31,32,33].

Throughout the Middle East, countries such as Egypt, Algeria, and Iran are projected to continue their status as significant consumers of wheat, characterized by high per capita consumption levels. Moreover, there is an optimistic outlook for global wheat-based ethanol production, with a rebound expected as rising production figures in India and China counterbalance the reduction observed in the European Union [34,35].

The EU produced 297.5 million tons of cereals in 2021, 12.1 million tons more than in 2020, rebounding from the drought-affected level of 2020 [36]. Europe is a major consumer of cereals, both for direct consumption and as animal feed. Wheat and maize are the most important cereals for human consumption in Europe, while barley is also commonly used for animal feed. In this global context, there an obvious need for protection measures for cereal crops.

Heavy metals have a significant impact on agricultural crops. Cereal crops, including wheat, rice, maize, barley, and oats, constitute a staple diet for millions worldwide. The consistent growth, quality, and yield of these crops are vital for ensuring food security. Unfortunately, the escalation of industrialization and anthropogenic activities, as mentioned above, has led to the accumulation of heavy metals in soils, which can be absorbed by plants, thereby impacting their growth, development, and nutritional quality [37,38]. The protection of cereal crops from heavy metal contamination is pivotal for safeguarding human health and global food security.

As a consequence of their toxicity, HMs can have detrimental effects on human health when they accumulate in edible parts of cereal crops. Cereals are a major source of dietary intake for various essential nutrients, including carbohydrates, proteins, vitamins, and minerals. However, when these crops are exposed to heavy metals, the toxic elements can be absorbed and accumulated in their tissues, posing a serious threat to consumers [39,40,41,42].

Cereals are commonly used to provide feed to animals, contributing to their growth, health, and overall productivity. Alfalfa (*Medicago sativa* L.) provides a high protein forage crop commonly fed to livestock including cattle, horses, or sheep and provides essential nutrients such as protein, fibre, vitamins, and minerals. Heavy metal contamination in alfalfa can lead to reduced growth and the accumulation of heavy metals such as cadmium, lead, or arsenic can affect the nutritional quality of the forage, affecting animal health. In addition, the consumption of alfalfa contaminated with heavy metals can lead to the accumulation of metals in animals’ tissues, as well as potential health problems. Also, maize (*Zea mays* L.), especially maize kernels, is a staple food for livestock, providing energy-rich carbohydrates and essential nutrients, and maize plants exposed to heavy metal-contaminated soil may show stunted growth, a lower yield, and reduced nutritional value. Heavy metals can interfere with nutrient uptake and lead to mineral imbalances in plants, affecting their overall quality as animal feed [43,44,45].

Careful monitoring of soil and plant health and the implementation of appropriate mitigation and remediation strategies are required.

### 1.3. Heavy Metal Impacts on Seed Germination for Cereals and Plants Used as Feed Sources for Livestock

Seed germination, the fundamental process by which a plant embryo transforms into a seedling, marks the inception of a plant’s life cycle. It is a critical event that influences the plant growth, crop yield, and ecosystem health. In recent years, the study of heavy metals and their impact on seed germination has gained significant attention. Heavy metals, such as lead, cadmium, mercury, and nickel, are natural constituents of the Earth’s crust, but have become increasingly prevalent due to anthropogenic activities. Understanding their effects on seed germination is imperative, as it directly influences plant establishment, crop production, and ecological balance. Therefore, seed germination is a critical stage in the life cycle of plants and serves as the first point of contact between seeds and the surrounding environment, including heavy metals present in the soil. The impact of heavy metals on seed germination can have significant implications for plant growth, crop yield, and the overall ecosystem [46].

Heavy metals can significantly impair seed germination, affecting plant growth, crop productivity, and ecosystem health. High concentrations of heavy metals, such as cadmium, lead, and mercury, can inhibit or delay the germination process. These metals can interfere with key physiological processes that are essential for seed germination, such as water uptake, enzyme activity, and cellular metabolism [47]. Moreover, heavy metals can disrupt water uptake by seeds during the imbibition process, where seeds absorb water and swell. This can lead to reduced water availability within the seed, hindering the activation of the metabolic processes necessary for germination [48].

Heavy metals can induce oxidative stress in seeds, causing an imbalance between the production of reactive oxygen species (ROS) and the antioxidant defense system. Excessive ROS production can damage cellular components, including DNA, proteins, and lipids, leading to impaired germination. Many enzymes are involved in the breakdown of the nutrients stored within seeds to provide energy for germination. Heavy metals can inhibit the activity of these enzymes, disrupting nutrient mobilization and energy production for seed germination [49,50].

Even if seeds manage to germinate in the presence of heavy metals, the subsequent radicle (embryonic root) and shoot growth can be compromised. This can result in stunted seedlings with reduced vigor and biomass. Seedlings that emerge from germinating seeds exposed to heavy metals may exhibit deformities, reduced root development, and altered leaf morphology. These effects can further limit the ability of plants to establish themselves and grow successfully [47,51,52].

The sensitivity of seeds to heavy metal toxicity varies among plant species and types of heavy metals. Some plant species may be more tolerant to specific heavy metals, while others are highly sensitive. Additionally, certain heavy metals may have a more severe impact on germination than others. The impact of heavy metals on seed germination can have long-term consequences for plant populations and ecosystem dynamics. Reduced germination rates can affect plant density, diversity, and succession patterns, leading to shifts in community composition [53,54].

It is important to note, however, that there exists considerable interspecific variation in the structure of seed coats. This diversity in seed coat morphology has a direct impact on the permeability of the seed coat. Seregin and Kozhevnikova (2005) investigated the permeability of the seed coat to three metals—Cd, Pb, and Ni—by examining how these metals were distributed within imbibing caryopses of *Zea mays* L. (commonly known as corn). Their findings revealed that cadmium and lead were confined to the cells of the seed coat; in contrast, nickel demonstrated the ability to traverse the seed coat and reach the endosperm and scutellum cells. Consequently, the permeability of the seed coat is also influenced by the distinct physical and chemical properties of various metals [48,55,56].

**Figure 2:**
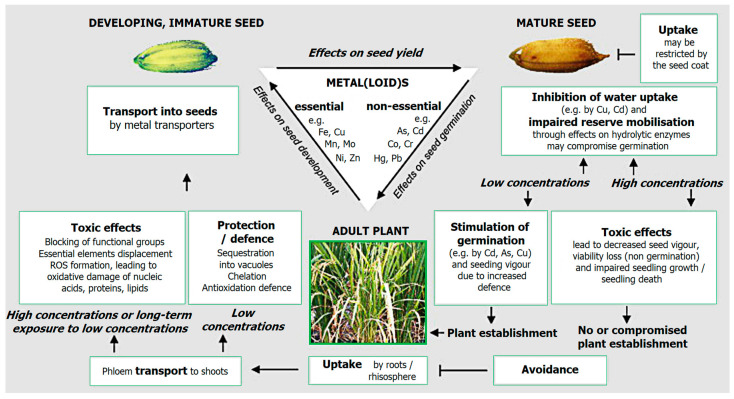
Simplified scheme summarizing our current knowledge of the effects of metal(loid)s on seeds; the images of Oryza sativa (rice) are used to represent seeds and plants generally. The effects on seed yield have not been widely studied, but the few studies available indicate that metal(loid) treatment of plants tends to decrease pod and seed yield [48], reproduced with Elsevier permission, License 5619301114983/31 August 2023).

Although the seed coat provides a measure of protection against metal-induced stress prior to germination, it eventually undergoes changes. These changes may involve cracking or an increase in permeability upon germination. The existing literature indicates that metals influence seed germination through two primary mechanisms. Firstly, metals exert their toxicity; secondly, they impede the uptake of water (Figure 2). Numerous research papers illustrate that exposing seeds to metal treatments results in a concentration-dependent decline in germination across a wide range of species. 

This emphasizes the significance of understanding how metal exposure can disrupt the germination process and subsequently impact plant growth and the overall ecosystem dynamics. Therefore, studying the impact of heavy metals on seed germination—in other words, of phytotoxicity—holds immense significance for various reasons, mainly referring to: agricultural productivity, ecological balance, environmental restoration, and human health. The study of heavy metals’ impact on seed germination is indispensable for comprehending the intricate interplay between anthropogenic activities, plant biology, and ecosystem dynamics. By deciphering the mechanisms through which heavy metals impede seed germination, researchers can develop strategies to mitigate their adverse effects and pave the way for healthier plant growth, sustainable agriculture, and the preservation of our natural environment. Thus, it is essential to emphasize the potential risks and consequences associated with heavy metal contamination in the environment, food chain, and livestock production, which can justify theoretical and experimental studies on the impact of heavy metals on seed germination for cereals and plants used as livestock feed sources, which is addressed in this paper.

The experimental study of the phytotoxicity of heavy metals on seed germination performed in this paper underscores the urgency of understanding their impacts on plant populations and ecosystems. As society grapples with environmental pollution and its consequences, efforts to assess, mitigate, and remediate heavy metal contamination are paramount. By comprehending the intricate interactions between heavy metals and seed germination, this paper can contribute to the protection of cereal and livestock-feeding plant crops.

## 2. Results and Discussion

### 2.1. Effects of Lead (Pb(II)) and Copper (Cu(II)) Metal Ions on Seed Germination and Seedling Development of Alfalfa (Medicago sativa L.) in a Soilless Growing Environment

#### 2.1.1. Influence of Pb(II) and Cu(II) Metal Ions on *Medicago sativa* L. Seeds Degree of Germination (G%)

Phytotoxicity tests were conducted in triplicate (10 seeds each), with *Medicago sativa* L. seeds initiating the germination process after a 24 h period. The germination phase was extended over 7 days, taking place in a sterile laboratory environment, and maintaining a temperature of 24 ± 2 °C.

**Figure 3:**
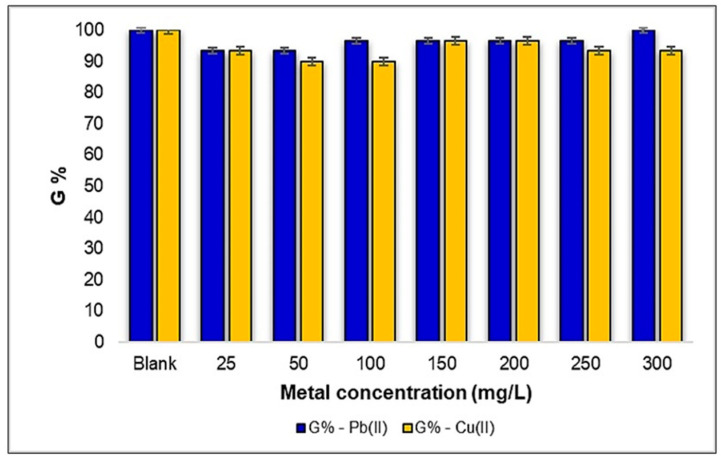
The influence of metal ions Pb(II) and Cu(II) on the degree of germination of a number of 30 seeds of *Medicago sativa* L.

The results obtained from the phytotoxicity tests revealed that the influence of Pb(II) and Cu(II) metal ions on the degree of germination in the *Medicago sativa* L. seeds, within the analyzed concentration range, exhibited a small effect compared to the control sample. Remarkably, this influence was characterized by consistent seed germination, with only minor percentage variations observed for both metal ions. Under the stress induced by Pb(II), the seed germination rates were observed to be 93% for concentrations of 25 and 50 mg/L and up to 100% for 300 mg/L. Conversely, under the influence of Cu(II) ions, the seeds germinated at a rate of 90% for concentrations between 50 and 100 mg/L, and up to 96% for concentrations between 150 and 200 mg/L. Consequently, although the alfalfa seed germination percentage was higher in the case of contamination with Pb(II) compared to Cu(II), the difference was a mere 3%. Notably, at a concentration of 300 mg Pb(II)/L, a striking phenomenon emerged: a 100% germination rate was recorded. This observation underscores the remarkable adaptability of *Medicago sativa* L. to thrive even under the stress imposed by Pb(II) and Cu(II) metal ions (Figure 3).

#### 2.1.2. Influence of Pb(II) and Cu(II) on the Growth and Development of *Medicago sativa* L.

**Figure 4:**
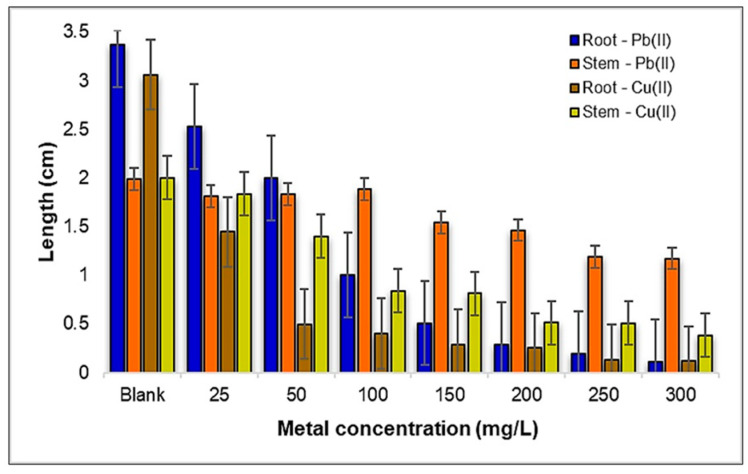
The influence of Pb(II) and Cu(II) metal ions on the development of *Medicago sativa* L. seedlings.

The data related to the influence of Pb(II) and Cu(II) on the growth and development of *Medicago sativa* L. reveal interesting insights into the impact of Cu(II) and Pb(II) concentrations on the growth of *Medicago sativa* L. Both the stem and root length exhibited a notable reduction as the Cu(II) concentrations increased, highlighting the potential toxic effects of metal ions on plant development, suggesting a correlation between the metal ion concentration and their harmful effects (Figure 4). 

**Figure 5:**
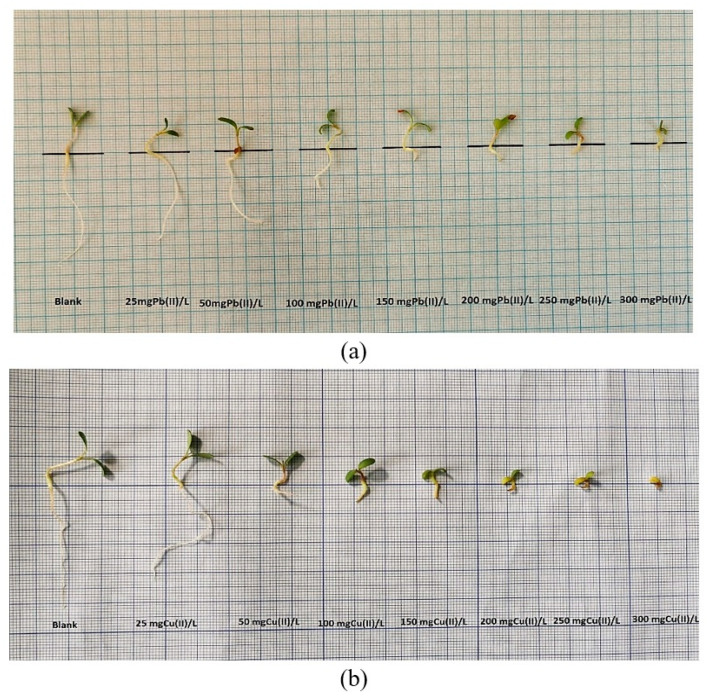
The effects of Pb(II) (**a**) and Cu(II) (**b**) on the growth of *Medicago sativa* L. seedlings.

Examining the effects of the Pb(II) concentration on *Medicago sativa* L., a consistent decrease in root length is evident as the Pb(II) concentration increases, with measurements ranging from 2.6 cm at 25 mg Pb(II)/L to a mere 0.12 cm at 300 mg Pb(II)/L. The stem length, however, displayed a varying pattern within the 25–100 mg Pb(II)/L concentration range, followed by a steadfast decrease beyond 150 mg Pb(II)/L, as illustrated in Figure 5a.

Shifting our focus to the influence of Cu(II) ions, it becomes apparent that the *Medicago sativa* L. experienced a significant reduction in both root and aerial development as the Cu(II) concentrations increased. The root length ranged from 1.45 cm at 25 mg Cu(II)/L to a mere 0.12 cm at 300 mg Cu(II)/L, while the stem length showed a similar trend, varying from 1.84 cm at 25 mg Cu(II)/L to 0.39 cm at 300 mg Cu(II)/L (Figure 5b).

When comparing the influence of the two metal ions on the growth of the *Medicago sativa* L. seedlings, improved development was observed under the stress induced by Pb(II). Consequently, under the influence of Pb(II) ions, the roots were approximately three times longer, and the stems about twice as long, as those contaminated with Cu(II) ions.

When comparing the effects of these two metals on the growth of *Medicago sativa* L., it becomes evident that Pb(II) metal ions tend to have a less detrimental impact, allowing for better overall plant development when contrasted with Cu(II). This observation underscores the distinct toxicity levels associated with different metal ions and their significant implications for plant growth.

#### 2.1.3. Evaluating Toxicity Levels and Metal Tolerance in the Roots and Stems of *Medicago sativa* L. Towards Pb(II) and Cu(II)

**Figure 6:**
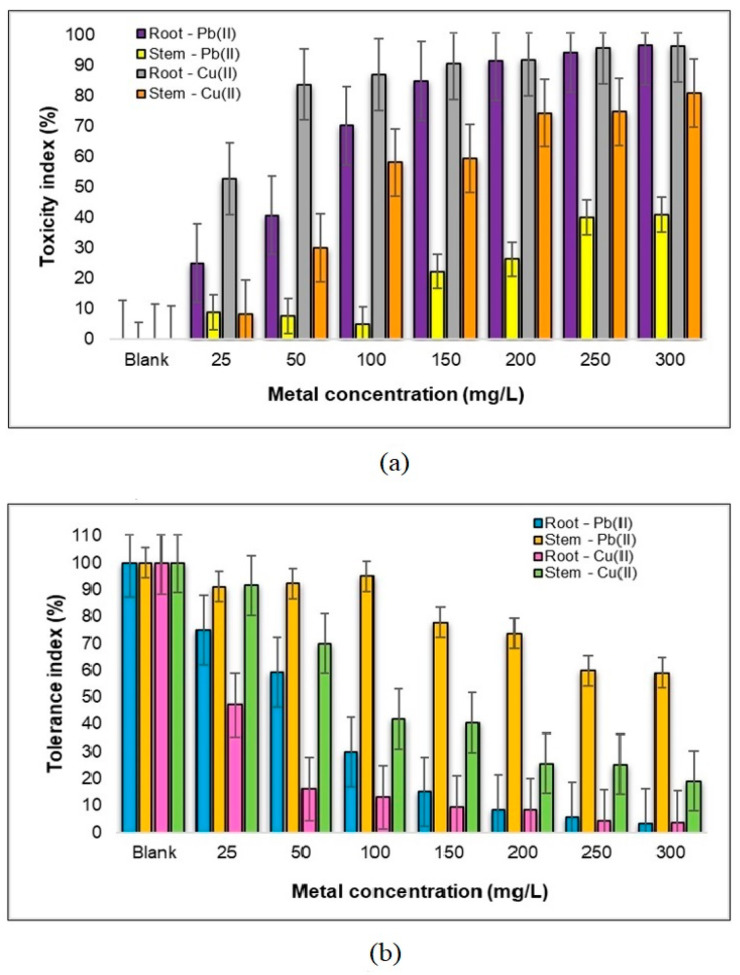
Toxicity index (**a**) and tolerance (**b**) of the components of a sample of 30 *Medicago sativa* L. seedlings to Pb(II) and Cu(II) metal ions.

Interesting insights emerge from the data presented in Figure 6, which delves into the toxicity (a) and tolerance (b) indices of the *Medicago sativa* L. plant when subjected to the influence of Pb(II) and Cu(II) metal ions. Firstly, in the presence of Pb(II) ions, an inhibition of seedlings below the 50% mark is evident, with the stems notably experiencing this restriction. As the Pb(II) concentration escalates to 150 mg Pb(II)/L, we observe a nearly equal level of toxicity affecting both the root and stem components of the plant. What is particularly interesting is the range of 25–50 mg/L, where the roots exhibit a striking double tolerance when compared to their response under the influence of Cu(II). This hints at a heightened ability of the root system to withstand the stress induced by Pb(II). Notably, even at a concentration as high as 100 mg Pb(II)/L, the roots show only a minimal 5% inhibition, underscoring their resilience.

In stark contrast, when confronted with Cu(II) stress, the *Medicago sativa* L. plant demonstrates significantly heightened sensitivity. Concentrations exceeding 200 mg/L result in an evident inhibition, exceeding 90% for roots and surpassing 70% for stems, indicating a severe adverse effect of Cu(II) ions on plant development. However, within the concentration range of 25–100 mg Cu(II)/L, the plant showcases a remarkable tolerance level, enduring the presence of these metal ions at a rate of approximately 95%. This highlights a stark divergence in the plant’s response to Cu(II) as opposed to Pb(II) ions.

Consequently, the data clearly indicate that the *Medicago sativa* L. plant has a higher tolerance to Pb(II) ions compared to Cu(II) metal ions, demonstrating a tolerance that is two times better in terms of the plant components. This disparity in tolerance levels highlights the distinct challenges posed by different heavy metal contaminants in the environment and underscores the plant’s ability to adapt to various metal stressors.

### 2.2. The Effects of Pb(II) and Cu(II) Metal Ions on the Growth and Development of Wheat (Triticum aestivum L.) in a Soilless Growing Environment

#### 2.2.1. Influence of Pb(II) and Cu(II) Metal Ions on the Germination Percentage (G%) of *Triticum aestivum* L. Seeds

**Figure 7:**
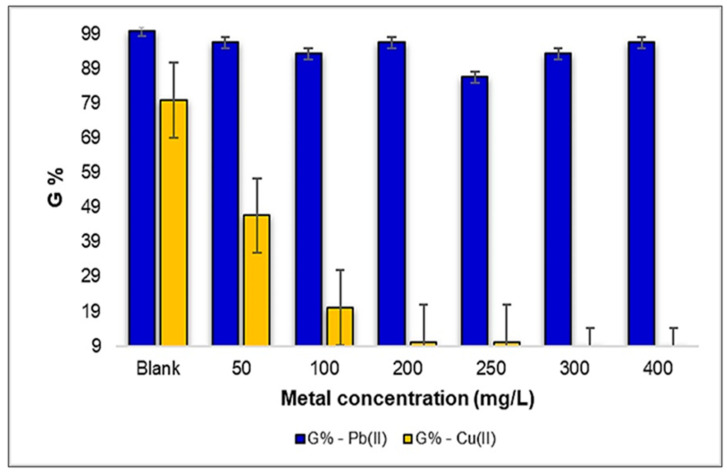
The influence of metal ions Pb(II) and Cu(II) on the degree of germination of a number of 30 seeds of *Triticum aestivum* L.

The phytotoxicity tests were conducted in triplicate, and the *Triticum aestivum* L. seeds initiated the germination process after an incubation period of 48 h. Under sterile laboratory conditions, with a controlled temperature of 24 ± 2 °C, the germination was monitored over a 5-day period. According to Figure 7, a noteworthy observation emerges regarding the germination rate of *Triticum aestivum* L. seeds in response to varying concentrations of Pb(II). Interestingly, it becomes evident that the germination rate remained relatively stable and unaltered when exposed to different Pb(II) concentrations, particularly in comparison to the control sample. Notably, at the concentration of 250 mg Pb(II)/L, the seed germination rate exhibited its lowest value, with a percentage of 87%. However, at concentrations of 50, 200, and 400 mg Pb(II)/L, the degree of germination closely mirrored that of the control sample, registering at 97%. This robust germination performance in the presence of Pb(II) suggests a notable resilience of *Triticum aestivum* L. seeds to this specific metal.

On the other hand, when investigating the influence of Cu(II) ions on the germination of *Triticum aestivum* L. seeds, a markedly contrasting representation emerges. As the Cu(II) concentration increases, we observe a pronounced reduction in the degree of germination. Notably, a significant drop of 10% is evident in the 200–250 mg Cu(II)/L concentration range, and a mere 4% reduction occurs within the 300–400 mg Cu(II)/L concentration range. These findings highlight a substantial inhibitory effect of Cu(II) ions on seed germination, particularly in the higher concentration ranges.

These results underscore the notable resistance of *Triticum aestivum* L. seeds to Pb(II) metal, with little impact on the germination rates, while emphasizing the heightened sensitivity of germination to Cu(II) ions, especially at higher concentrations. Comparing the two metals, a 70% higher germination rate of *Triticum aestivum* L. seeds was observed under the influence of Pb(II) ions compared to Cu(II). These findings reveal the varying responses of plants to different heavy metal contaminants, which have important implications for understanding their ecological interactions and adaptation strategies in metal-contaminated environments.

#### 2.2.2. Influence of Pb(II) and Cu(II) Metal Ions on the Growth and Development of *Triticum aestivum* L.

**Figure 8:**
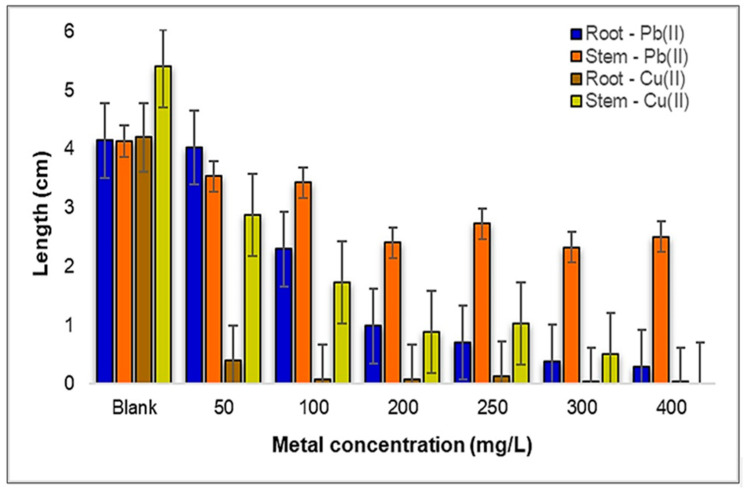
Influence of Pb(II) and Cu(II) metal ions on the development of *Triticum aestivum* L. seedlings.

In the concentration range depicted in Figure 8, it is observed that the growth rate of the roots is consistently slower than that of the stems as the concentration increases to 100 mg/L. Interestingly, it is worth noting that at the concentration of 50 mg Pb(II)/L, there is an encouraging observation of improved root development compared to the control sample. However, as we scrutinize the data further, a significant reduction in the root lengths becomes evident with the increasing metal concentrations, both for Pb(II) and Cu(II). This indicates that heavy metal exposure, particularly within the concentration range of 300–400 mg Pb(II)/L, exerts a pronounced inhibitory effect on root growth. This observation underscores the vulnerability of the root system to elevated metal concentrations, which can have implications for nutrient uptake and overall plant health.

**Figure 9:**
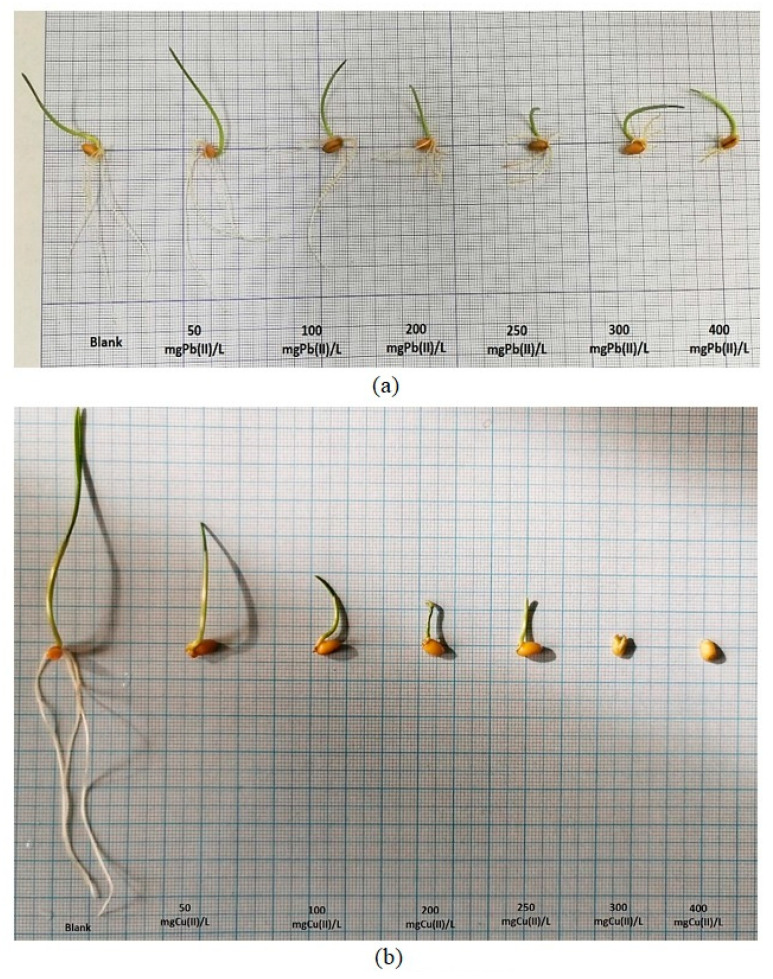
The effects of (**a**) Pb(II) and (**b**) Cu(II) on the growth of *Triticum aestivum* L. seedlings.

Shifting our focus to the development of the stems, a consistent decreasing trend is observed across the selected concentration range shown in Figure 9a. Remarkably, the concentration of 300 mg Pb(II)/L demonstrates the lowest growth rate of the stems when compared to the control sample. This decline in stem growth underlines the sensitivity of this plant species to higher Pb(II) concentrations, potentially impacting its above-ground biomass and overall vigor.

When examining the influence of Cu(II) ions on the *Triticum aestivum* L. seedlings, a strikingly reduced root development is noted, particularly at the concentration of 50 mg Cu(II)/L, where the root length is a mere 0.4 cm. This inhibitory effect becomes even more pronounced, with root lengths diminishing to just 0.03 cm at the higher concentrations of 300–400 mg Cu(II)/L. In contrast, concerning the aerial part of the seedlings, development is only observed up to the concentration of 300 mg Cu(II)/L, as depicted in Figure 9b. This disparity between root and stem development in response to Cu(II) stress highlights the disproportionate impact on different plant parts, further emphasizing the complexity of metal–plant interactions.

Comparing the influence of the two metal ions on the growth of the *Triticum aestivum* L. seedlings, enhanced development was observed under the stress induced by Pb(II). Consequently, under the influence of Pb(II) ions, the roots are approximately three times larger, and the stems about twice as long, as those contaminated with Cu(II) ions.

Despite the evident decrease in both the root and stem growth rates with the increasing metal concentrations, it is noteworthy that the *Triticum aestivum* L. seeds managed to exhibit some degree of development, even at concentrations as high as 400 mg/L. This resilience underscores the remarkable adaptability and survival mechanisms employed by this plant species when faced with challenging heavy metal environments.

#### 2.2.3. Toxicity Levels and Tolerance of the Root and Stem of *Triticum aestivum* L. to Pb(II) and Cu(II) Metals

**Figure 10:**
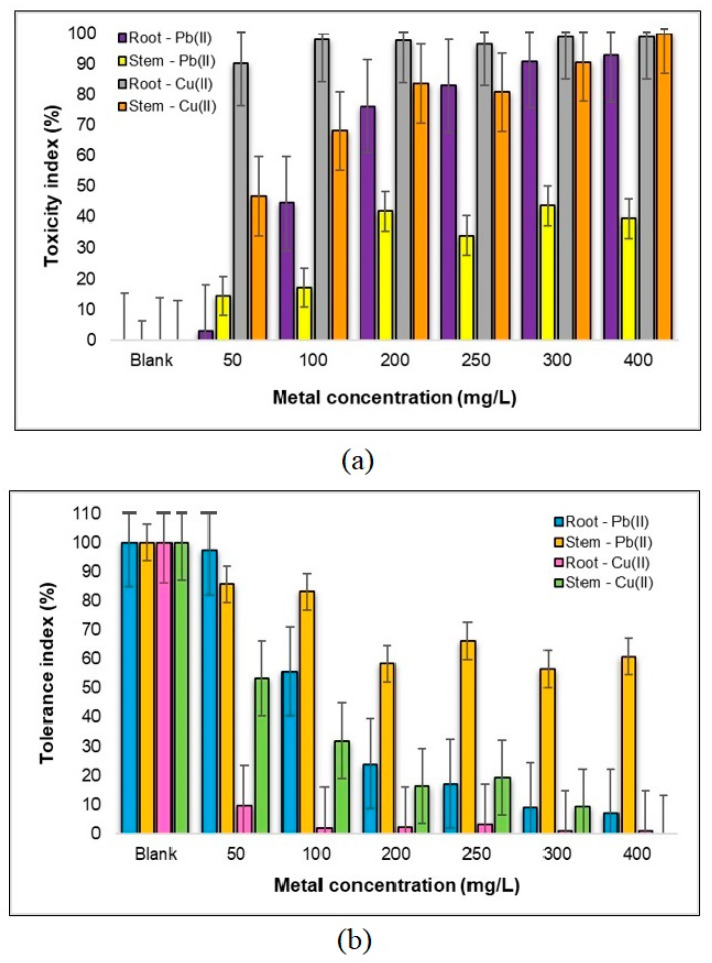
Toxicity index (**a**) and tolerance (**b**) of the components of a sample of 30 *Triticum aestivum* L. seedlings to Pb(II) and Cu(II) metal ions.

In the context of the toxicity index highlighted in Figure 10, we can discern some interesting patterns and insights concerning the impact of the different metal concentrations on the roots and stems of the *Triticum aestivum* L. plants. Starting with the roots, a clear trend emerges, indicating that the toxicity increases as we move across the selected concentration range. At the lower end, the concentration of 50 mg Pb(II)/L exhibits no significant toxic effect, showcasing a good tolerance level of nearly 100% to Pb(II) metal ions. Even at 100 mg Pb(II)/L, we observe only a tolerable level of metal toxicity. However, as we progress into the concentration range of 200–400 mg Pb(II)/L, we note a substantial negative impact on root development, with the toxicity percentages rising as high as 93%. This indicates that the root system is particularly vulnerable to the influence of higher Pb(II) concentrations.

Turning our attention to the stems, a dissimilar pattern emerges. The toxic effects of the Pb(II) solution do not exhibit a straightforward linear increase with higher metal concentrations. Instead, there appears to be a fluctuation, characterized by both increasing and decreasing toxicity levels. Intriguingly, the concentration of 50 mg Pb(II)/L showcases the lowest toxicity on the stems, measuring just 14%. However, within the concentration range of 300–400 mg Pb(II)/L, the root system of *Triticum aestivum* L. demonstrates a remarkably low tolerance, dipping below 10%. The tolerance of the plant stems exhibits fluctuation as well. In the range of 50–100 mg Pb(II)/L, there is a gradual decrease of approximately 15%, transitioning from 86% to 83%. Subsequently, in the range of concentrations from 100–200 mg Pb(II)/L, there is an almost twofold decrease in tolerance, representing a drop of about 30%. This intricate interplay between toxicity and tolerance underscores the complex and varied responses of different plant components to Pb(II) contamination.

Focusing on the graphs in Figure 10 related to Cu(II) metal ions, we observe a starkly different scenario. The root system appears to be significantly inhibited, with toxicity percentages ranging between 90% (at 50 mg Cu(II)/L) and 99% (in the 300–400 mg Cu(II)/L range). In contrast, the stems exhibit their highest tolerance at the concentration of 250 mg Cu(II)/L, at approximately 20%. However, the stems display a remarkable sensitivity to Cu(II) stress, with a complete inhibitory effect observed at the concentration of 400 mg Cu(II)/L.

These findings show the complex and varying responses of different plant parts to both Pb(II) and Cu(II) metal ions. While roots are particularly sensitive to higher Pb(II) concentrations, the stem responses exhibit fluctuations in the toxicity levels. Furthermore, Cu(II) metal ions appear to exert a notably inhibitory effect on the root system, highlighting the complex dynamics of heavy metal–plant interactions in metal-contaminated environments.

Based on the obtained results, it can be concluded that the inhibitory effect of Cu(II) ions demonstrates a notably enhanced magnitude, being 2-fold higher in the case of roots and 3-fold higher for stems, in comparison to the inhibitory impact exerted by Pb(II) ions on *Triticum aestivum* L.

#### 2.2.4. Influence of Pb(II) and Cu(II) on the Total Biomass of *Triticum aestivum* L.

**Figure 11:**
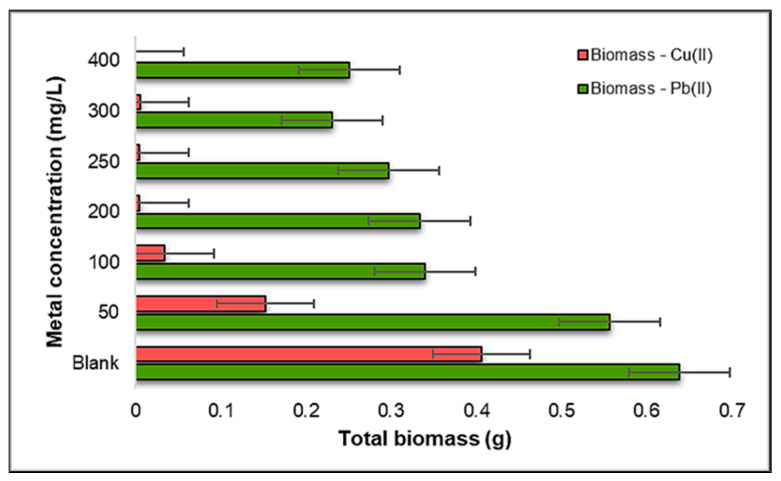
Influence of Pb(II) and Cu(II) metal ions on the biomass of *Triticum aestivum* L. seedlings.

Following the measurement of the plant components, the harvested biomass underwent a drying process in an oven, maintaining a temperature of 105 °C for a duration of 15 h. Subsequently, the dried biomass was weighed after allowing it to cool. The result of these steps provides valuable insights into the total biomass (comprising both roots and stems) for each concentration of the two metals, as depicted in Figure 11.

In light of the results presented earlier, a striking pattern emerges with respect to the total biomass of the *Triticum aestivum* L. seedlings under the influence of Cu(II) ions. Notably, there is a significantly lower biomass observed, underscoring the profound inhibitory effect of this metal on the development of the selected plant species. This finding emphasizes the critical role that metal toxicity plays in shaping the overall growth and vitality of these seedlings.

However, it is worth noting a particularly interesting observation in the context of Pb(II) exposure. At a concentration of 50 mg Pb(II)/L, there is a noticeable increase in the biomass contaminated with Pb(II), reaching almost twice the amount at 0.64 g, compared to that contaminated with Cu(II), which is 0.4 g. However, at the highest concentration examined in the study—specifically, 400 mg Pb(II)/L—the biomass contaminated with Pb(II) ions significantly reduces, to 0.25 g. This finding underscores a notable tolerance of *Triticum aestivum* L. seedlings to Pb(II) stress at lower concentrations, allowing for more robust biomass accumulation.

These findings highlight the intricate relationship between heavy metal concentrations and the resultant biomass of *Triticum aestivum* L. seedlings. While Cu(II) exerts a significant inhibitory effect, leading to reduced biomass, the influence of Pb(II) is more nuanced, with lower concentrations surprisingly resulting in enhanced biomass production, five times higher than Cu(II). These insights contribute to our understanding of how different heavy metals impact plant development, with potential implications for the ecological dynamics of metal-contaminated environments.

### 2.3. Effects of Pb(II) and Cu(II) on the Development of Corn (Zea mays L.) in a Soilless Growing Environment

#### 2.3.1. Influence of Pb(II) and Cu(II) on the Degree of Germination of *Zea mays* L. Seeds (G%)

In this experimental series, the seeds of the chosen plant species entered the germination process 72 h after their preparation. This germination process was conducted accurately, with all germinated seeds in Petri dishes being counted and the average calculated across all three replicates for each concentration used. These values were then compared to those of the control sample, which was prepared under identical experimental conditions but did not contain the toxic heavy metal solutions of Pb(II) or Cu(II).

**Figure 12:**
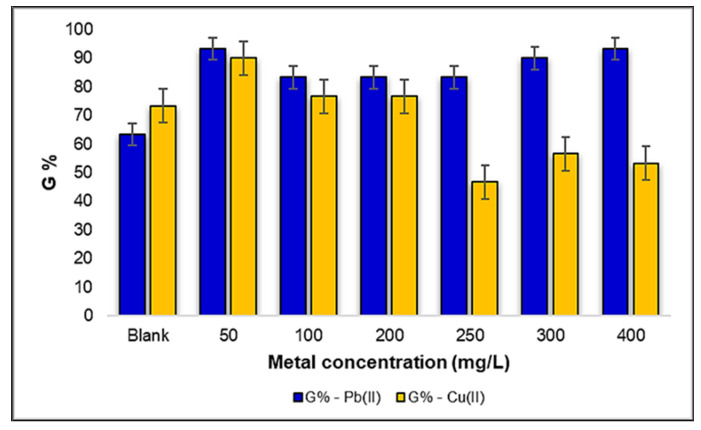
The influence of metal ions Pb(II) and Cu(II) on the degree of germination of a number of 30 seeds of *Zea mays* L.

When delving into the results obtained after germination, as depicted graphically in Figure 12, several interesting patterns and effects appear. Firstly, within the selected concentration range of 50–400 mg Pb(II)/L, it becomes evident that the seed germination rate surpasses that of the control sample. In the absence of heavy metal exposure, the control sample exhibited a germination rate of only 63%. However, the highest degree of germination among the *Zea mays* L. seeds was recorded at the concentrations of 50 and 400 mg Pb(II)/L, with the germination rates reaching an impressive 93%. This intriguing observation highlights a stimulating effect of Pb(II) ions on the germination of *Zea mays* L. seeds, a finding that warrants further investigation to understand the underlying mechanisms at play.

Conversely, the results indicate a contrasting trend in seed germination when subjected to the stress generated by Cu(II) in the same concentration range. While the maximum percentage of germination was highlighted at the concentration of 50 mg Cu(II)/L, reaching an impressive 90%, the influence of Cu(II) ions led to a lower germination rate at higher concentrations, notably dropping to a minimum of 47% at 250 mg Cu(II)/L. Intriguingly, as the concentrations increased to 300–400 mg Cu(II)/L, there was no significant inhibitory effect observed on the seeds of *Zea mays* L., with the germination maintaining a percentage of approximately 57%. This intricate interplay between Cu(II) stress and seed germination underscores the complex nature of heavy metal–plant interactions and the differing responses of plant species to varying metal stressors.

Based on the obtained results, it was found that the germination rate of the *Zea mays* L. seeds under the stress induced by Pb(II) increased by approximately 17% compared to the seeds under the influence of Cu(II) metal ions.

These results not only reveal the contrasting effects of Pb(II) and Cu(II) ions on the germination of *Zea mays* L. seeds, but also highlight the potential stimulatory impact of Pb(II) ions within a certain concentration range. These observations prompt further research into the mechanisms underlying these effects and their ecological implications in metal-contaminated environments.

#### 2.3.2. Influence of Pb(II) and Cu(II) on the Growth and Development of *Zea mays* L.

**Figure 13:**
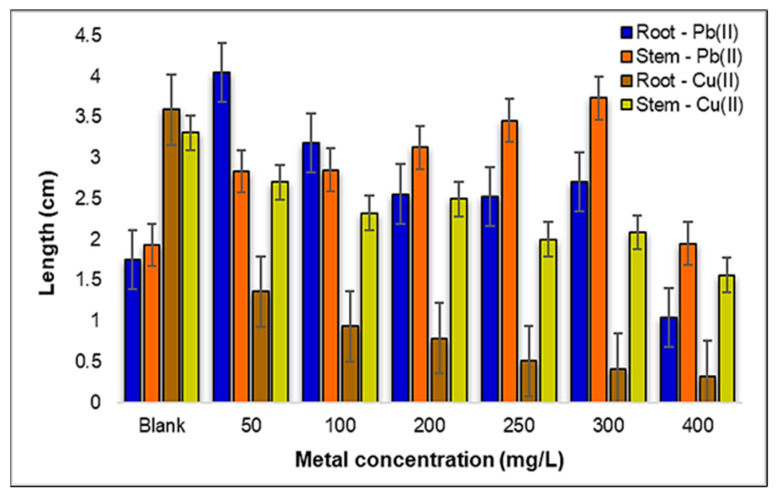
Influence of Pb(II) and Cu(II) metal ions on the development of *Zea mays* L. seedlings.

In this phase of the experiment, all of the developed *Zea mays* L. seedlings were carefully collected, and their root and stem lengths were precisely measured using a ruler. These measurements were meticulously recorded in centimeters and represent the average values obtained from the three replicates, as graphically depicted in Figure 13.

**Figure 14:**
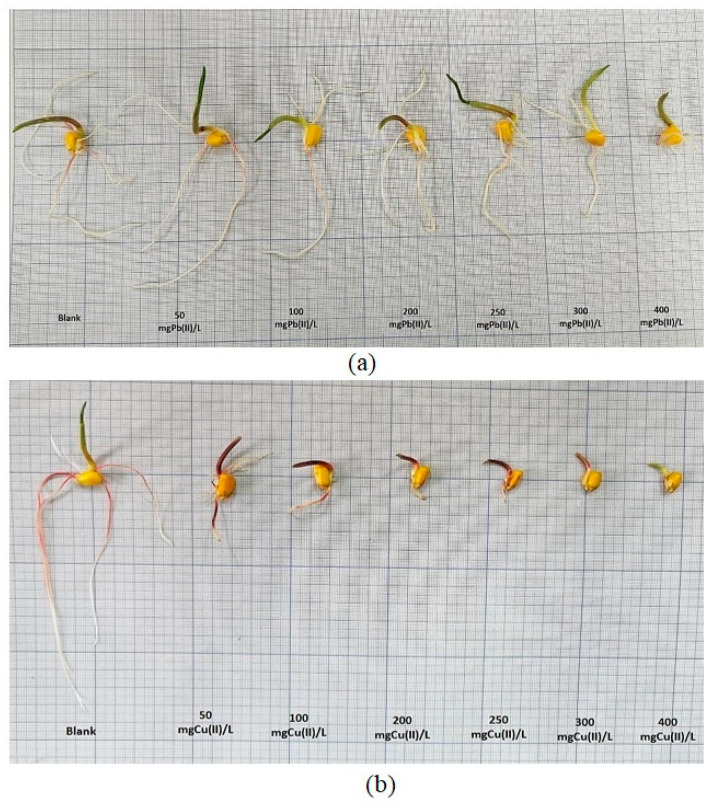
Effects of (**a**) Pb(II) and (**b**) Cu(II) on the growth of *Zea mays* L. seedlings.

Analyzing the influence of Pb(II) ions on the development of both the roots and stems of *Zea mays* L., as illustrated in Figure 14a, the results offer interesting insights. Within the concentration range of 50–100 mg Pb(II)/L, a notable trend occurs: the rate of root elongation surpasses that of stem development. However, as we move into the concentration range of 200 to 400 mg Pb(II)/L, a noticeable shift arises, and the development of the root system becomes less robust compared to that of the stems. This observation suggests that, under specific conditions, the roots of *Zea mays* L. can exhibit more pronounced activity and growth than their above-ground counterparts; namely, the stems.

This phenomenon has significant implications, particularly in the context of phytoremediation processes, where plant roots play a crucial role in metal uptake and detoxification. Shifting our focus to the impact of Cu(II) metal ions, a different pattern develops.

The growth rate of the plant strains remains relatively constant within the selected concentration range, indicating a degree of resilience to Cu(II) stress. However, the growth of the root system begins to show sensitivity to Cu(II) ions with an increase in the concentration, particularly noticeable at 250 mg Cu(II)/L. At the concentration of 400 mg Cu(II)/L, the rate of root growth drops drastically, reaching a level approximately 12 times lower than that of the control sample. Nevertheless, it is worth noting that *Zea mays* L. demonstrates a remarkable capacity to develop even under the influence of substantial Cu(II) metal stress, as illustrated in Figure 14b.

These results not only reveal the contrasting effects of Pb(II) and Cu(II) ions on the development of *Zea mays* L., but also highlight the remarkable adaptability of this plant species to challenging environments. It can be mentioned that corn roots contaminated with Pb(II) show double growth compared to those contaminated with Cu(II) metal. Additionally, the observation that roots can exhibit greater activity than stems in certain metal concentrations underscores the intricate and dynamic nature of plant responses to heavy metal exposure, which holds relevance for phytoremediation strategies and environmental management.

#### 2.3.3. The Degree of Toxicity and the Tolerance of the Root and Stem of *Zea mays* L. to the Pb(II) and Cu(II)

**Figure 15:**
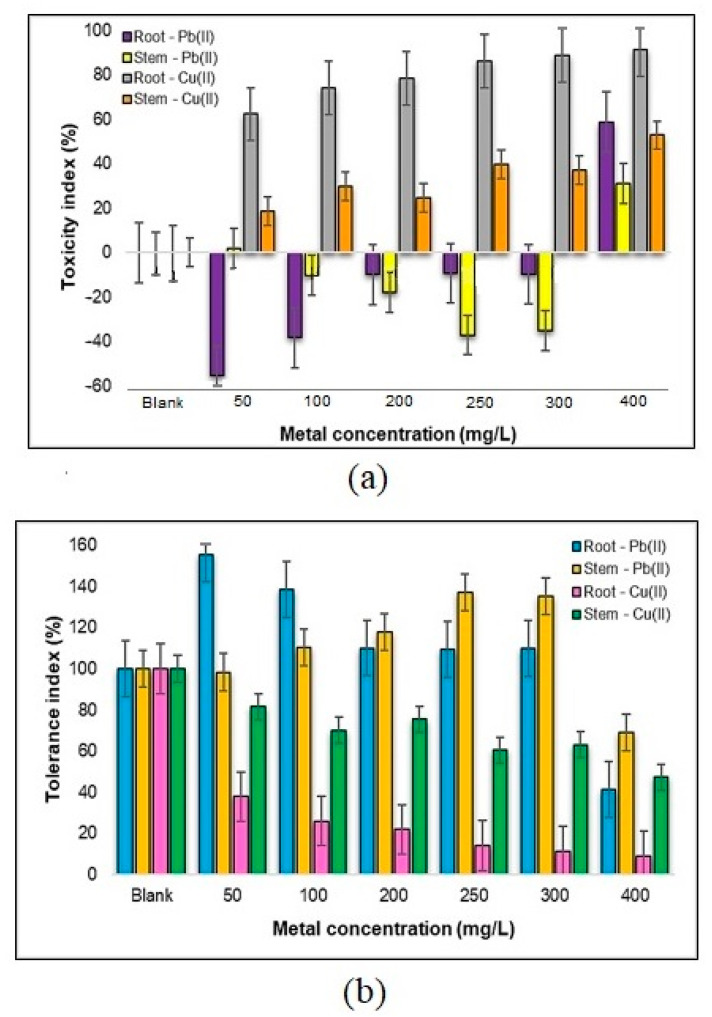
Toxicity index (**a**) and tolerance (**b**) of the components of a sample of 30 *Zea mays* L. seedlings to Pb(II) and Cu(II) metal ions.

Analyzing the data presented graphically in Figure 15, we can consider several assumptions on the tolerance of *Zea mays* L. plant components to the presence of heavy metal ions, specifically Pb(II) and Cu(II). These observations provide a comprehensive understanding of how these plant parts respond to varying metal concentrations and offer valuable insights into their adaptability.

In terms of the tolerance to Pb(II) ions, it is evident that the tolerance of the plant components undergoes a notable decrease as the concentration of Pb(II) ions increases, although a uniform, constant decrease is not observed. Within the range of 50–100 mg Pb(II)/L, the highest tolerance is displayed by the roots, indicating their resilience to these metal ions. In contrast, the concentration of 400 mg Pb(II)/L yields considerably lower tolerance levels, with the roots exhibiting a tolerance of 41.4% and the stems at 50.9%. The tolerance index of the *Zea mays* L. seedlings demonstrates a progressive decrease within the concentration intervals of 50–100 mg Pb(II)/L and 250–400 mg Pb(II)/L. An interesting difference is observed at the concentration of 200 mg Pb(II)/L, where the tolerance is slightly lower than that at 250 mg Pb(II)/L. This discrepancy might be attributed to external factors, such as variations in the light conditions or seed quality. The overall tolerance index of the *Zea mays* L. seedlings to Pb(II) showcases a substantial decline, falling from 253.79% at 50 mg Pb(II)/L to 92.39% at 400 mg Pb(II)/L, as depicted in Figure 15a.

In terms of the sensitivity to Cu(II) ions, it was found that, in stark contrast to the response to Pb(II) ions, a significant inhibition of the *Zea mays* L. seedlings is noted with the increasing Cu(II) concentration. At the highest concentration tested (400 mg Cu(II)/L), the root growth experiences a substantial 91% inhibition, while the stem growth is inhibited by 53%. Within the concentration range of 50–300 mg Cu(II)/L, the plant displays a considerable tolerance of over 60% for the stems, indicating a degree of resilience to Cu(II) stress at these levels. However, in the case of roots, the percentage of tolerance decreases progressively with the increase in the Cu(II) concentration. This can be attributed to the fact that the root system is the first point of contact with the metal, and as the concentrations rise, the inhibitory effects become more pronounced (Figure 15b).

These outcomes provide critical insights into the dynamic response of *Zea mays* L. plant components to Pb(II) and Cu(II) metal ions. While the roots exhibit remarkable tolerance to Pb(II) at certain concentrations, they are highly sensitive to Cu(II) ions, showing a difference of about six times lower. Meanwhile, the stems demonstrate resilience to Cu(II) within a certain range, but display overall inhibition with the increasing concentrations. These observations contribute to our understanding of plant–metal interactions and have implications for phytoremediation strategies and environmental management in metal-contaminated areas.

#### 2.3.4. Influence of Pb(II) and Cu(II) on the Total Biomass of *Zea mays* L.

**Figure 16:**
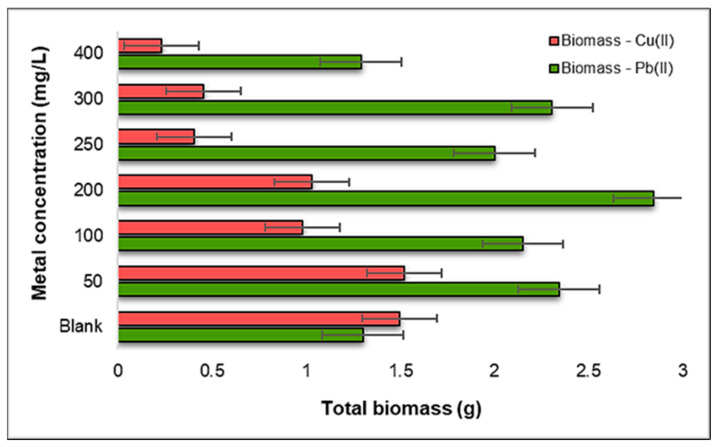
The influence of Pb(II) and Cu(II) metal ions on the biomass of *Zea mays* L.

Examining the total biomass of the *Zea mays* L. seedlings obtained after the experiment, as presented in Figure 16, we observe that, contrary to conventional expectations, a higher biomass is apparent in the seedlings developed under the influence of Pb(II) metal ions compared to those exposed to Cu(II) ions. This observation is particularly remarkable given that Pb(II) ions are typically considered more toxic than Cu(II) ions, indicating a complex interplay between metal toxicity and plant growth responses.

In the case of contamination with Pb(II) ions, the plant demonstrates robust growth and development, with a higher biomass compared to the control sample observed in the concentration range of 50–300 mg Pb(II)/L. This suggests that *Zea mays* L. seedlings are notably resilient and capable of thriving even when exposed to moderate levels of Pb(II). Interestingly, the increase in the Pb(II) concentration to 400 mg Pb(II)/L does not result in a substantial decrease in biomass. This finding implies that, within this tested range, the plant maintains its growth and development despite higher levels of Pb(II) exposure.

On the contrary, in the case of Cu(II) contamination, a distinct pattern emerges. The total biomass of the *Zea mays* L. seedlings remains relatively stable up to the concentration of 200 mg Cu(II)/L, indicating a degree of resilience to Cu(II) stress at these levels. However, as we move into the concentration range of 250–400 mg Cu(II)/L, a significantly lower biomass is consistently observed compared to the control sample. This sharp decline in biomass suggests that the plant is notably affected by higher Cu(II) concentrations, and its growth and development are considerably hampered under such conditions.

These results challenge the conventional expectations regarding the toxic effects of Pb(II) and Cu(II) ions on *Zea mays* L. seedlings. Despite Pb(II) being considered more toxic, the plant displays better growth and development under its influence in a certain concentration range. This complex interplay between metal toxicity and plant response underscores the need for a nuanced understanding of plant–metal interactions. Additionally, the distinct responses to Cu(II) contamination, where higher concentrations lead to reduced biomass—two times lower than the biomass contaminated with Pb(II) ions—highlight the sensitivity of *Zea mays* L. to elevated Cu(II) levels. These insights have significant implications for understanding plant adaptation to heavy metal contamination in diverse environmental settings.

### 2.4. Significance of Results

The study highlights the diverse responses of these three plant species to Pb(II) and Cu(II) ions, emphasizing their importance not only as crops, but also in selecting appropriate plants for phytoremediation based on the specific metal contaminants present in a contaminated site. Understanding these responses can aid in the development of effective and sustainable strategies for the remediation of metal-contaminated environments. Further research is needed to unravel the underlying mechanisms governing these plant–metal interactions and to optimize phytoremediation techniques.

The remarkable adaptability of *Medicago sativa* L. to Pb(II) and Cu(II) ions suggests that this plant species could serve as a potential candidate for phytoremediation in metal-contaminated soils. Its high germination rates even at moderately elevated metal concentrations indicate its ability to establish and propagate in such environments. While both Pb(II) and Cu(II) affect root and stem growth, the lesser impact of Pb(II) implies that *Medicago sativa* L. could potentially withstand moderate Pb(II) contamination without severe growth inhibition. This adaptability could be beneficial in remediating soils contaminated with lead. The higher tolerance to Pb(II) ions, particularly in the root system, suggests that its roots could potentially play a vital role in the absorption and detoxification of lead in contaminated soils. This property may make it a valuable candidate for phytoremediation efforts targeted at lead contamination.

*Triticum aestivum* L. resistance to Pb(II) ions during germination suggests its suitability for cultivation in soils with moderate lead contamination. However, its sensitivity to Cu(II) ions, especially at higher concentrations, implies that it may not perform well in copper-contaminated environments. Wheat’s ability to maintain some degree of growth, even at high metal concentrations, is noteworthy. This resilience might be beneficial in situations where soil contamination cannot be entirely avoided. While wheat’s roots exhibit sensitivity to higher Pb(II) concentrations, its stems display fluctuations in sensitivity, making it an interesting candidate for further study to understand the differential impact on root and stem development. For Cu(II) contamination, the plants’ sensitivity in both its roots and stems at high concentrations indicates limitations in its tolerance.

*Zea mays* L. displays unique responses to both Pb(II) and Cu(II) ions. The stimulation of germination by Pb(II) ions within a specific concentration range may indicate its potential for use in environments with moderate lead contamination. The ability of *Zea mays* L. to develop even at high concentrations of both metals suggests its resilience to heavy metal stress. This adaptability can be advantageous in soils with varying metal concentrations. The differential sensitivity of its roots and stems to Pb(II) and Cu(II) ions highlights the complexity of metal–plant interactions in corn. The pronounced sensitivity of its roots to Cu(II) indicates that corn may not be ideal for Cu(II) phytoremediation.

## 3. Materials and Methods

### 3.1. Framework for the Application of Phytotoxicity Studies

Traditionally, the focus of environmental assessment has primarily centered on chemical characterization, particularly regarding heavy metals. This emphasis has led to the formulation of guidelines aimed at guiding environmental protection regulations. However, these parameters fall short in providing insights into the dynamic phytotoxicity essential for ascertaining the bioavailability of contaminants within an ecosystem [57].

Recognizing the necessity for environmental management rooted in the outcomes of toxicological assessments marked a pivotal step in pollution control. Numerous endeavors have been undertaken to devise rationales and methodologies for environmental measurement and remediation. Consequently, the concept of ecosystem health has emerged within the realm of environmental toxicology. Within this framework, chemical characterization, phytotoxicity assessment, and ecological evaluation draw upon a diverse array of methods to address the entire biological community within an ecosystem [57].

Phytotoxicity tests find numerous applications, but, in general, they serve as a valuable environmental tool by providing insights into:

- Identifying and ranking the phytotoxicity potential of samples;
- Defining the actual degree of phytotoxicity;
- Pinpointing the chemical components within a complex waste mixture contributing to phytotoxicity (referred to as phytotoxicity evaluation or TIE—phytotoxicity identification evaluation).

From all these applications, it becomes evident that the primary advantage of bioassays lies in their ability to furnish data on heavy metals’ phytotoxicity in plants. Nevertheless, their principal drawback is the inability to pinpoint the specific substance responsible for the observed effects. Consequently, phytotoxicity assessment must be complemented with chemical characterization.

In alignment with this initial advantage, phytotoxicity tests serve to directly quantify the overall phytotoxicity of a sample without necessitating the individual identification and analysis of each pollutant. The topic of phytotoxicity assessment remains a subject of debate. Nonetheless, the use of phytotoxicity tests allows for the direct measurement of the potential environmental impacts of various heavy metals, providing information that is otherwise unattainable through alternative means.

### 3.2. Indicators of Heavy Metals Phytotoxicity in Plants

In this section, we introduce the key indicators employed to evaluate the phytotoxicity of heavy metals in plants, specifically focusing on their impact on seed germination and seedling development, which serve as sensitive and informative indicators of heavy metal contamination in soil and water. These stages are critical junctures in a plant’s life, where the initial exposure to contaminants can have profound and lasting effects.

#### 3.2.1. The Degree of Germination

The germination criterion is defined by the emergence of the cotyledon or the appearance of a radicle measuring at least 2 mm in length. Seeds that do not exhibit these specific characteristics by the end of the designated development period will not be considered when calculating the germination degree (*G*), calculated according to Equation (1) [58,59].

(1) $$G\left(\%\right)=\frac{S_{germ}}{M_{germ}}\times 100$$

where:

*G* is the degree of germination (%);
*S_germ_* is the number of germinated seeds in the sample to be analyzed (the sample containing heavy metal solutions of different concentrations);
*M_germ_* is the number of germinated seeds in the control sample.

#### 3.2.2. Relative Growth of Plant Root and Stem Lengths

Determining this indicator entails measuring the lengths of the roots, stems, and leaves of each plant and calculating the average length for each individual plant component. Subsequently, the relative growth in length for each plant component (root, stem, and leaf) is calculated by comparing it to the control sample, using Equation (2) [60].

(2) $$G_{r}=\frac{L_{average}}{L_{blank}}\times 100$$

#### 3.2.3. Growth Inhibition Index of Plant Components (*El*%)

To assess the phytotoxicity of heavy metals on the growth of various plant components, Equation (3) can be applied to calculate the growth inhibition index [61].

(3) $$El\left(\%\right)=\frac{Blank\;root/stem\;length\;(\mathrm{cm})-Sample\;root/steem\;length\;(\mathrm{cm})}{Sample\;root/stem\;length\;(\mathrm{cm})}\times 100$$

#### 3.2.4. Tolerance Index of Plant Components (*Er*%)

In order to determine the tolerance index of plants to heavy metals, Equation (4) is usually applied [62].

(4) $$Er(\%)=\frac{Average\;root/stem\;sample\;length\;(\mathrm{cm})}{Root\;length/control\;stem\;(\mathrm{cm})}\times 100$$

### 3.3. Biological Material

For developing the phytotoxicity testing, corn (*Zea mays* L., Pioneer P9537 hybrid), wheat (*Triticum aestivum* L., GLOSSA variety), and alfalfa (*Medicago sativa* L.) were chosen as the standard species for experimentation. These plant species were selected due to their suitability for laboratory cultivation, their low nutritional demands, and their rapid growth, typically germinating within 5–7 days.

Maize and wheat together contribute significantly to global cereal production, accounting for approximately 80% of it. Moreover, maize, wheat, and alfalfa have been extensively studied by researchers over time for their potential in phytoremediation. They have demonstrated tolerance to heavy metals, exhibiting high bioaccumulation factors, rapid growth rates, and substantial biomass yield per hectare [63].

Considering these factors, our research focused on assessing the tolerance of these selected plant species to the phytotoxicity of Pb(II) and Cu(II) ions.

### 3.4. Working Method

Corn, wheat, and alfalfa plant species were used in the experimental phytotoxicity tests, along with PbCl_2_ and CuCl_2_ solutions. These experiments were conducted in triplicate, with 10 mL of metal solutions of varying concentrations placed in Petri plates containing Whatman filter paper. The concentration of the metal solutions ranged between 25 and 400 mg/L. Each plate held 10 seeds of each plant species, while sterile water was used for the control sample. The experiments took place in a sterile laboratory environment at a temperature of 24 ± 2 °C. The Petri plates were covered and left at room temperature, alternating between light and dark conditions, to initiate the germination and plant growth processes, spanning a period of 5 and 7 days.

Prior to the experiments, the seeds underwent sterilization. This involved washing them with 99% ethyl alcohol for 20 s, followed by immersion in a 20% sodium hypochlorite solution for 10 min. Subsequently, the seeds were dried in an oven for 24 h at a temperature of 27 °C. The Petri plates were also sterilized through autoclaving for 20 min at 121 °C and 1 atm pressure, followed by 24 h of drying at 35 °C in an oven. After germination, the growth of plantlets was evaluated for each concentration of heavy metal solution by measuring the primary plant components; namely, the root and stem length. The inhibitory effect of the contaminants was determined by comparing the test groups with the control groups. Various parameters were assessed, including the degree of germination of wheat seeds, the elongation rate of seedling components, the phytotoxicity index, and the tolerance index for roots and stems, as well as the quantity of green and dry biomass. The measurement results were recorded in centimeters, and the conditions for conducting the phytotoxicity test are summarized in Table 1.

## 4. Conclusions

This comprehensive paper explores the significant impact of heavy metals on the environment, agriculture, and food security. It highlights the persistence of heavy metals in the environment and their accumulation in ecosystems, posing risks to both wildlife and human populations. The paper discusses how heavy metals contaminate soil through natural and anthropogenic processes, leading to various adverse effects on plant growth and crop yields. It also emphasizes the importance of understanding the sources and effects of heavy metals, especially in the context of global cereal production and consumption.

The paper further examines the detrimental effects of heavy metals on cereal crop growth and quality, elucidating their role in global food security. It emphasizes the need for protection measures for cereal crops and presents the data on global cereal utilization and consumption patterns. It also discusses the potential long-term effects of heavy metal contamination on arable land, emphasizing the economic implications and trade barriers associated with contaminated cereals. Furthermore, the work examines the impact of heavy metals on plants used as feed sources for livestock. It underscores how heavy metal contamination can compromise the growth, nutritional quality, and safety of these plants, ultimately affecting animal health and productivity.

The discussion extends to the critical phase of seed germination, highlighting how heavy metals can inhibit or delay this process, impacting plant growth, crop yield, and ecosystem dynamics. The paper emphasizes the importance of studying heavy metals’ phytotoxicity and its significance for agricultural productivity, ecological balance, and human health.

Further, this study investigates the impact of Pb(II) and Cu(II) metal ions on the germination and development of three different plant species—namely *Medicago sativa* L. (alfalfa), *Triticum aestivum* L. (wheat), and *Zea mays* L. (corn)—in a soilless growing environment. The research encompassed various aspects, including seed germination, root and stem growth, toxicity, and tolerance to heavy metal exposure, as well as total biomass production.

For *Medicago sativa* L., the results indicated that both Pb(II) and Cu(II) metal ions had limited effects on the seed germination within the analyzed concentration range. Remarkably, at 300 mg Pb(II)/L, a 100% germination rate was observed, showcasing the plant’s adaptability to metal stress. However, both metals negatively affected the growth of the plant, with Cu(II) exerting a more substantial inhibitory effect on the root and aerial development compared to Pb(II).

In *Triticum aestivum* L., the study revealed that Pb(II) had little impact on the seed germination, while Cu(II) showed a dose-dependent inhibitory effect. Interestingly, Pb(II) enhanced the root growth at lower concentrations but inhibited it at higher levels, while Cu(II) consistently suppressed root development as the concentrations increased. Stem growth was also negatively affected by both metals.

*Zea mays* L. exhibited a surprising response to Pb(II) exposure, with enhanced seed germination rates within a specific concentration range. Cu(II), on the other hand, had a dose-dependent inhibitory effect on the germination. The root and stem growth in the *Zea mays* L. showed distinct patterns under Pb(II) and Cu(II) stress, with Pb(II) exhibiting a more complex influence and Cu(II) causing significant inhibition at higher concentrations.

The toxicity and tolerance indices revealed that *Medicago sativa* L. had higher tolerance to Pb(II) than Cu(II). *Triticum aestivum* L. demonstrated sensitivity to Cu(II) stress, while *Zea mays* L. exhibited varying responses to both metals.

Remarkably, the *Zea mays* L. displayed higher biomass under Pb(II) influence in a certain concentration range, challenging conventional expectations. In contrast, Cu(II) contamination led to reduced biomass, highlighting the plant’s sensitivity to elevated Cu(II) levels.

Overall, this study provides valuable insights into the diverse and dynamic responses of different plant species to Pb(II) and Cu(II) metal ions, shedding light on their adaptability and resilience in metal-contaminated environments. It emphasizes the complexity of metal–plant interactions and their ecological implications.

These findings can be valuable for understanding how different plants adapt to heavy metal contamination in various environmental settings and for informing phytoremediation strategies in metal-contaminated soils. Understanding these impacts can help in developing strategies to mitigate the adverse effects of heavy metal contamination and the need for research, monitoring, and mitigation strategies to address these issues and to safeguard both environmental and human well-being.

Further research is needed to uncover the underlying mechanisms driving these responses and to develop effective remediation techniques. These findings have important implications for understanding plant–metal interactions and devising phytoremediation strategies in contaminated ecosystems.

## Figures and Tables

**Table 1:** Process conditions for the evaluation of germination inhibition and seedling development tests.

Plant Species	*Zea mays* L., *Triticum aestivum* L., *Medicago sativa* L.
Incubation temperature	24 ± 2 °C.
Photo-periodicity	12 h of light/12 h of darkness.
Type of test vessel	Petri dishes with a diameter of 10 cm.
Number of seeds/plate	10.
The number of replicates	3 replicates for each concentration to be tested.
Duration of the test	5–7 days.
Compounds containing Pb(II) and Cu(II) used to prepare aqueous solutions	PbCl_2_ and CuCl_2_: 50 mg/L, 100 mg/L, 200 mg/L, 250 mg/L, 300 mg/L, 400 mg/L (for corn and wheat); 25 mg/L, 50 mg/L, 100 mg/L, 150 mg/L, 200 mg/L, 250 mg/L, 300 mg/L (for alfalfa).
Biological measurements	The degree of germination, the length of the stems, the length of the roots.
Reported parameters	The degree of germination of cereal seeds (G, %) in the presence of heavy metals compared to the control sample; Root length (cm)—average of seedlings from one plate and average of the 3 replicates; Stem length (cm)—average of seedlings from one plate and average of the 3 replicates; Toxicity index (%) of heavy metals on the root and stem of seedlings; Tolerance index (%) of root and seedling stem for heavy metal.
Equipment used	Radwag five-decimal analytical balance; Multiparameter Schott Instruments proLab 2000, Mainz, Germany; Raypa autoclave; Niche Biobase Smoke Hood, Model: FH1000(A).

## Data Availability

The data presented in this study are available in the graphs and tables provided in the manuscript.
